# Buprenorphine Dose and Time to Discontinuation Among Patients With Opioid Use Disorder in the Era of Fentanyl

**DOI:** 10.1001/jamanetworkopen.2023.34540

**Published:** 2023-09-18

**Authors:** Laura C. Chambers, Benjamin D. Hallowell, Andrew R. Zullo, Taylor J. Paiva, Justin Berk, Rachel Gaither, Aidan J. Hampson, Francesca L. Beaudoin, Rachel S. Wightman

**Affiliations:** 1 Department of Epidemiology, Brown University, Providence, Rhode Island; 2 Substance Use Epidemiology Program, Rhode Island Department of Health, Providence; 3 Department of Medicine, Alpert Medical School of Brown University, Providence, Rhode Island; 4 Division of Therapeutics and Medical Consequences, National Institute on Drug Abuse, National Institutes of Health, Bethesda, Maryland; 5 Department of Emergency Medicine, Alpert Medical School of Brown University, Providence, Rhode Island

## Abstract

**Importance:**

Buprenorphine treatment for opioid use disorder (OUD) has more than doubled since 2009. However, current US Food and Drug Administration buprenorphine dosing guidelines are based on studies among people using heroin, prior to the emergence of fentanyl in the illicit drug supply.

**Objective:**

To estimate the association between buprenorphine dose and time to treatment discontinuation during a period of widespread fentanyl availability.

**Design, Setting, and Participants:**

This retrospective cohort study used statewide Rhode Island Prescription Drug Monitoring Program data. Participants were Rhode Island residents initiating buprenorphine treatment for OUD between October 1, 2016, and September 30, 2020. Data analysis was performed from December 9, 2022, to August 10, 2023.

**Exposure:**

Daily dose of buprenorphine (16 mg and 24 mg) defined starting on the day of initiation based on total quantity and days’ supply dispensed. Patients were censored on any dose change.

**Main Outcomes and Measures:**

Buprenorphine treatment discontinuation in the 180 days following initiation, defined as a gap in treatment of more than 27 days based on prescription fill dates and days’ supply. Kaplan-Meier and Cox regression survival analyses were conducted to estimate the association between buprenorphine dose and time to treatment discontinuation, controlling for potential informative censoring and measured potential confounders.

**Results:**

Among 6499 patients initiating buprenorphine treatment for OUD, most were aged 25 to 44 years (57%; n = 3682), were male (61%; n = 3950), and had private (47%; n = 3025) or Medicaid (33%; n = 2153) insurance. More than half of patients were prescribed a daily dose of interest at initiation (16 mg: 50%; n = 3264; 24 mg: 10%; n = 668). In Kaplan-Meier analyses, 58% of patients discontinued buprenorphine treatment within 180 days (16 mg: 59% vs 24 mg: 53%; log-rank test *P* = .005). In Cox regression analyses, patients prescribed a dose of 16 mg had a greater risk of treatment discontinuation than those prescribed 24 mg (adjusted hazard ratio, 1.20; 95% CI, 1.06-1.37).

**Conclusions and Relevance:**

In this cohort study of patients initiating buprenorphine treatment from 2016 to 2020, patients prescribed a 24 mg dose of buprenorphine remained in treatment longer than those prescribed 16 mg. The value of higher buprenorphine doses than currently recommended needs to be considered for improving retention in treatment.

## Key Points

**Question:**

Are higher doses of buprenorphine treatment for opioid use disorder associated with improved retention in treatment when use of fentanyl (vs heroin) is more prevalent?

**Findings:**

In this cohort study of 6499 patients initiating buprenorphine treatment between 2016 and 2020, those prescribed the recommended daily dose (16 mg) were at significantly greater risk of treatment discontinuation within 180 days than those prescribed a higher dose (24 mg).

**Meaning:**

The results of this study suggest that the value of higher buprenorphine doses than currently recommended needs to be considered for improving retention in treatment.

## Teaser

This cohort study estimates the association between buprenorphine dose and time to treatment discontinuation in patients with opioid use disorder during a period of widespread fentanyl availability.

## Introduction

Fentanyl and other potent synthetic opioids (hereafter referred to as *fentanyl*) are driving overdose deaths in the US, with more than 71 000 fentanyl-related deaths in 2021 alone.^1^ Buprenorphine is effective for treatment of opioid use disorder (OUD) and is known to decrease mortality risk by 50% and confer other health and social benefits.^2,3,4,5,6,7^ The US Food and Drug Administration (FDA) recommends a target dose of 16 mg for buprenorphine maintenance treatment, with an upper limit of 24 mg.^8,9,10^ This dosing guidance was established prior to the emergence of fentanyl in the illicit drug supply^2^ and has not been formally reevaluated since fentanyl became widely available.

Some physicians have suggested that the current daily maintenance target dose of buprenorphine (16 mg) may be inadequate to control withdrawal and cravings in patients who used fentanyl^11^ and that a higher daily dose may better suppress withdrawal and cravings.^12,13,14^ Such opinions are consistent with case studies demonstrating benefits of higher buprenorphine doses in patients who used fentanyl.^15,16^ Preclinical studies have shown that fentanyl is more efficacious as an agonist of mu opioid receptors than morphine,^17^ and so it downregulates opioid receptor expression to a higher degree.^18^ Correspondingly, fentanyl also induces tolerance to a higher degree than morphine, a result of reduced opioid receptor expression.^19^ Together, these findings perhaps explain why substitution therapy with a low-efficacy agonist like buprenorphine may require higher doses when treating patients exposed to fentanyl than those exposed to morphinan opioids (eg, heroin and oxycodone). Although observational analyses have also suggested higher buprenorphine doses may potentially improve treatment effectiveness,^20,21,22,23,24,25,26,27,28^ the findings have not been confirmed in a large study using data from the fentanyl era that considers patients’ daily dose throughout follow-up.

To better understand the effectiveness of buprenorphine doses in the era of fentanyl for treatment of OUD, we conducted a retrospective cohort study to estimate the association between patients’ daily buprenorphine dose and retention in treatment over 180 days. The study was conducted in a statewide population of Rhode Island residents initiating buprenorphine treatment during a period with widespread fentanyl availability (2016 to 2020).^29^ During this period, fentanyl was involved in most unintentional overdose deaths in Rhode Island, with 59% in 2016 and increasing to 76% in 2020.^30^ We hypothesized that, if fentanyl use increases the optimal buprenorphine dose, patients prescribed a lower dose would be more likely to discontinue treatment within 180 days of initiation.

## Methods

### Study Data, Design, and Sample

We used data from the Rhode Island Prescription Drug Monitoring Program (PDMP) to conduct a retrospective cohort study of residents initiating buprenorphine treatment for OUD for the first time between October 1, 2016, and September 30, 2020. We aimed to ensure that patients were new to buprenorphine treatment by including data from April 1, 2016, when complete PDMP data became available. Each patient was followed for up to 180 days after initiation to evaluate retention in treatment (ie, including PDMP data through March 31, 2021). The study was approved by the Brown University and Rhode Island Department of Health Institutional Review Boards. Given that the study used administrative PDMP data, patients did not provide informed consent for this study. This study followed the Strengthening the Reporting of Observational Studies in Epidemiology (STROBE) reporting guideline.

Our analysis was limited to sublingual buprenorphine formulations indicated for OUD treatment (ie, sublingual buprenorphine/naloxone film or tablets and buprenorphine monoproducts). Products specifically FDA-approved for pain management were excluded (branded forms and generic equivalents). We also excluded patients dispensed injectable buprenorphine formulations or other alternative formulations in the 180 days after treatment initiation due to dosing differences and small sample sizes.

### Key Measures

#### Outcome

The study outcome was time until buprenorphine treatment discontinuation during the 180-day period following initiation. Discontinuation was defined as a gap in treatment of more than 27 days based on prescription fill dates and days’ supply. Patients who filled prescriptions early (ie, before finishing the days’ supply from the prior prescription) were credited the extra days on their subsequent prescription to reflect total days’ supply. The treatment discontinuation date was defined as the final day of the available supply or final day of the available supply prior to a gap of more than 27 days during the study period. Patients still engaged in treatment after 180 days were censored. Each patient’s study follow-up period was defined as the time from treatment initiation to treatment discontinuation or censoring, whichever occurred first.

#### Exposure

The study exposure was the patient’s daily dose of buprenorphine, defined starting on the day of initiation based on the total quantity and days’ supply dispensed for their first prescription. Patients were censored if/when their daily dose changed. We categorized daily dosing to account for slight variations in prescribing: 2 mg (0 to <3 mg), 4 mg (3 to <6 mg), 8 mg (6 to <10 mg), 12 mg (10 to <14 mg), 16 mg (14 to <18 mg), 20 mg (18 to <22 mg), 24 mg (22 to <26 mg), 28 mg (26 to <30 mg), and 32 mg (≥30 mg). Our primary analyses considered 2 dose categories: 16 mg and 24 mg, which are the recommended and upper limit daily doses on the FDA-approved package insert, respectively.^8,9,10^ In exploratory analyses, we also considered an 8 mg dose; however, given current dosage guidance, this was not a primary focus. We were unable to consider daily doses of more than 24 mg due to the small number of patients prescribed such doses during the study period.

#### Baseline Measures

Baseline patient sociodemographic characteristics were identified a priori and defined based on the first buprenorphine prescription: age group (18 to 24, 25 to 34, 35 to 44, 45 to 54, or ≥55 years), sex assigned at birth (female, male, or unknown), health insurance type (Medicaid, Medicare, private, or other or none), year of buprenorphine treatment initiation (2016, 2017, 2018, 2019, or 2020), and distance from home to pharmacy based on zip code centroids (<5 miles, ≥5 miles, or unknown). Additionally, we considered whether patients had an available days’ supply of a prescription benzodiazepine or opioid (other than buprenorphine medication for OUD) in the prior 30 days at baseline. These measures were hypothesized to be associated with censoring due to a dose change and potentially confound the association between daily dose and time to treatment discontinuation. The PDMP database does not include information on race and ethnicity.

### Statistical Analysis

Study data were analyzed using SAS version 9.4 (SAS Institute). Statistical significance was set at 2-sided *P* < .05. We compared the sociodemographic and prescription characteristics of patients by initial daily dose category (16 vs 24 mg) using χ^2^ tests. We used Kaplan-Meier and Cox regression survival analyses to estimate the association between daily dose and time to treatment discontinuation, controlling for potential informative censoring with stabilized inverse probability of censoring weights and controlling for potential confounding using stabilized inverse probability of treatment (16 mg vs 24 mg) weights. In Kaplan-Meier analyses, we compared the time to treatment discontinuation by daily dose using a log-rank test. Inverse probability of censoring weights and inverse probability of treatment weights were calculated based on baseline age group, sex assigned at birth, health insurance type, year of treatment initiation, distance from home to pharmacy, any available days’ supply of a benzodiazepine in the prior 30 days, and any available days’ supply of an opioid in the prior 30 days. Cox regression models were fit with robust SEs.

Two stability analyses were used to assess whether our results were robust to certain decisions about the study design and analysis. First, to consider the potential impact of patients who never initiated buprenorphine treatment, we excluded patients who had only been dispensed 1 buprenorphine prescription and/or a total of less than 7 days’ supply in the 180 days after treatment initiation. Second, given that titration up to a stable dose typically occurred within 30 days of treatment initiation, we also conducted an analysis restricted to patients engaged in treatment for at least 30 days, defining their daily dose category on day 30 following initiation (if they did not have a prescription that covered day 30, then their most recent prior dose was used) and starting follow-up on day 30.

## Results

**Figure 1.**
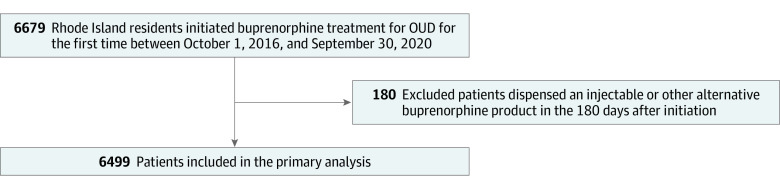
Flowchart of Study Inclusion and Exclusion Criteria OUD indicates opioid use disorder.

From October 1, 2016, to September 30, 2020, 6679 Rhode Island residents initiated buprenorphine treatment for OUD for the first time (Figure 1). Of those, 180 patients (3%) were excluded from the study because they received an injectable or other alternative buprenorphine formulation. The remaining 6499 patients were included in the study.

### Characteristics of the Study Sample

Overall, most patients were aged 25 to 44 years (57%; n = 3682), were male (61%; n = 3950), and had private (47%; n = 3025) or Medicaid (33%; n = 2153) health insurance (Table 1). The initial daily buprenorphine dose for most patients was 8 mg (21%; n = 1343) or 16 mg (50%; n = 3264). Only 10% of patients (n = 668) were initially prescribed 24 mg, and 0.2% (n = 15) were initially prescribed more than 24 mg. Most patients received a film formulation (58%; n = 3747), started with a supply of less than 8 days (58%; n = 3760), and lived less than 5 miles from their pharmacy (72%; n = 4711).

**Table 1.** Characteristics of the Study Sample

Characteristic	Participants, No. (%)			*P* value
	Overall (N = 6499)	Initial daily dose^a^		
		16 mg (n = 3264)	24 mg (n = 668)	
Sociodemographic characteristics				
Age group, y				
18-24	503 (8)	254 (8)	33 (5)	<.001
25-34	1966 (30)	1044 (32)	185 (28)	
35-44	1716 (26)	912 (28)	195 (29)	
45-54	1206 (19)	599 (18)	128 (19)	
≥55	1108 (17)	455 (14)	127 (19)	
Sex assigned at birth				
Female	2434 (37)	1182 (36)	239 (36)	.004
Male	3950 (61)	1987 (61)	424 (63)	
Unknown	115 (2)	95 (3)	5 (1)	
Health insurance type				
Medicaid	2153 (33)	1162 (36)	214 (32)	.03
Medicare	622 (10)	282 (9)	77 (12)	
Private	3025 (47)	1555 (48)	311 (47)	
Other or none	699 (11)	265 (8)	66 (10)	
Year of treatment initiation				
2016	543 (8)	285 (9)	57 (9)	.01
2017	1963 (30)	1090 (33)	217 (32)	
2018	1776 (27)	904 (28)	151 (23)	
2019	1355 (21)	605 (19)	140 (21)	
2020	862 (13)	380 (12)	103 (15)	
Prescription history in prior 30 d				
Available days’ supply of a benzodiazepine				
Yes	990 (15)	448 (14)	119 (18)	.006
No	5509 (85)	2816 (86)	549 (82)	
Available days’ supply of an opioid other than buprenorphine				
Yes	1072 (16)	475 (15)	125 (19)	.006
No	5427 (84)	2789 (85)	543 (81)	
Initial buprenorphine prescription characteristics				
Product type				
Buprenorphine monoproduct	406 (6)	142 (4)	48 (7)	.002
Buprenorphine/naloxone	6093 (94)	3122 (96)	620 (93)	
Product formulation				
Film	3747 (58)	1887 (58)	392 (59)	.68
Tablet	2752 (42)	1377 (42)	276 (41)	
Days’ supply, d				
<8	3760 (58)	1951 (60)	334 (50)	<.001
≥8	2739 (42)	1313 (40)	334 (50)	
Distance from home to pharmacy (miles)^b^				
<5	4711 (72)	2303 (71)	494 (74)	.16
≥5	1760 (27)	941 (29)	172 (26)	
Unknown	28 (<1)	20 (1)	<5^c^	

^a^ Defined as the daily dose on their first buprenorphine prescription, based on the total quantity and days’ supply dispensed.

^b^ Based on zip code centroids.

^c^ Counts of 1 to 4 and associated percentages are suppressed in accordance with the Small Numbers Policy of the Rhode Island Department of Health.

In bivariate analyses comparing patients prescribed an initial daily dose of 16 mg and 24 mg, their sociodemographic characteristics and 30-day prescription history differed. Patients prescribed 16 mg more often were younger (40% vs 33% were aged 18 to 34 years), had Medicaid insurance (36% vs 32%), and had initiated buprenorphine treatment in 2016 to 2018 (70% vs 64%), while they less often were male (61% vs 63%) and had recent prescriptions for benzodiazepines (14% vs 18%) and opioids (15% vs 19%). In contrast, initial buprenorphine prescription characteristics for patients prescribed 16 mg and 24 mg were similar in terms of product formulation (film vs tablet) and distance from their home to the pharmacy. However, patients prescribed an initial daily dose of 16 mg were more often prescribed a buprenorphine/naloxone product (96% vs 93%) and an initial days’ supply of less than 8 days (60% vs 50%).

### Treatment Discontinuation

**Figure 2.**
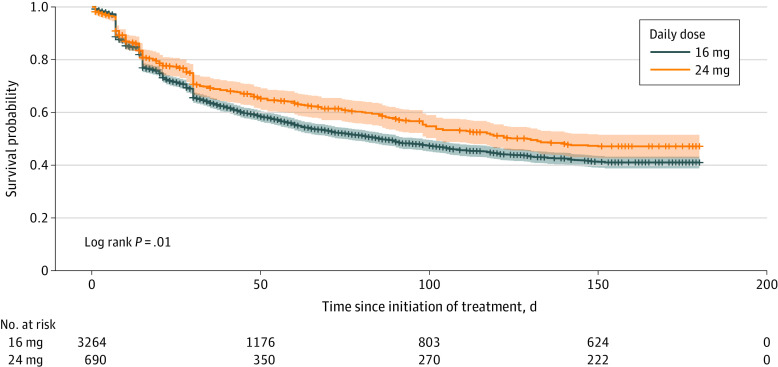
Time to Buprenorphine Treatment Discontinuation in the 180 Days After Initiation, by Daily Dose Starting on Day 0 (Primary Analysis) Treatment discontinuation is defined as a gap in treatment of more than 27 days, based on prescription fill dates and days’ supply. Analysis controls for potential informative censoring using stabilized inverse probability of censoring weights and for potential confounding using stabilized inverse probability of treatment weights. Survival probability indicates the probability patients are retained in buprenorphine treatment, with the shaded area representing the 95% CI. The number of patients at risk over time has been reweighted and, thus, is not expected to align with the overall study sample size.

During the 180-day follow-up period, 46% of patients (n = 2960) were censored due to a dose change. Patients initially prescribed 16 mg were more likely to be censored due to a dose change than those prescribed 24 mg (39% vs 26%; χ^2^ test *P* < .001). Among patients prescribed 16 mg and 24 mg who were censored due to a dose change, 71% (n = 902) and 90% (n = 156), respectively, had experienced a dose increase, and the median time to the dose change was 15 days (IQR, 7-42 days) and 20 days (IQR, 7-54 days), respectively. In Kaplan-Meier analyses with censoring due to dose changes and reweighting to control for potential informative censoring and confounding, 59% of patients initially prescribed 16 mg discontinued buprenorphine treatment within 180 days compared with 53% of those prescribed 24 mg (log-rank test *P* = .005) (Figure 2). This difference was also seen in Cox regression analyses with censoring due to dose changes and reweighting to control for potential informative censoring and confounding. Patients prescribed 16 mg were more likely to discontinue treatment than those prescribed 24 mg (adjusted hazard ratio, 1.20; 95% CI, 1.06-1.37) (Table 2). In stability analyses which excluded patients dispensed only 1 prescription and/or a total of less than 7 days’ supply during follow-up and restricted to patients engaged in treatment for at least 30 days and considering the daily dose prescribed on day 30 (eTable, eFigure 1 in Supplement 1), the results were similar to the primary analysis. In exploratory analyses, time to treatment discontinuation among patients prescribed 8 mg and 16 mg was similar (eFigure 1, eFigure 2 in Supplement 1).

**Table 2.** Association Between Daily Dose of Buprenorphine and Time to Treatment Discontinuation in the 180 Days After Initiation^a^^,^^b^

Daily dose	Hazard ratio (95% CI)	
	Unadjusted	Adjusted
**Primary analysis: defining daily dose and starting follow-up on day 0^c^**		
16 mg	1.22 (1.07-1.38)	1.20 (1.06-1.37)
24 mg	1 [Reference]	1 [Reference]
**Stability analysis: defining daily dose and starting follow-up on day 0^d^**		
16 mg	1.22 (1.05-1.42)	1.18 (1.01-1.37)
24 mg	1 [Reference]	1 [Reference]
**Stability analysis: defining daily dose and starting follow-up on day 30^e^**		
16 mg	1.18 (1.02-1.37)	1.16 (1.00-1.34)
24 mg	1 [Reference]	1 [Reference]

^a^ Each model controls for potential informative censoring using stabilized inverse probability of censoring weights and for potential confounding using stabilized inverse probability of treatment weights.

^b^ Defined as a gap in treatment of more than 27 days, based on prescription fill dates and days’ supply.

^c^ Limited to patients who started buprenorphine treatment at 16 mg or 24 mg, and censoring patients if/when they deviated from that dose.

^d^ Limited to patients who were dispensed more than 1 buprenorphine prescription and at least 7 total days’ supply during follow-up and started buprenorphine treatment at 16 mg or 24 mg, and censoring patients if/when they deviated from that dose.

^e^ Limited to patients who engaged in buprenorphine treatment for at least 30 days and were prescribed 16 mg or 24 mg on day 30, and censoring patients if/when they deviated from that dose.

## Discussion

During a period when fentanyl was common in the illicit drug supply in Rhode Island,^29^ nearly 5 times as many patients with OUD were initially prescribed a buprenorphine daily dose of 16 mg compared with 24 mg. However, those prescribed the higher dose were 20% more likely to be retained in treatment over 180 days. Of those prescribed 16 mg buprenorphine, 59% discontinued treatment within 180 days compared with 53% of those prescribed 24 mg. Patients prescribed an initial daily dose of 24 mg were more likely than those prescribed 16 mg to have initiated treatment in later years (eg, 2019 and 2020).

We interpret the findings as evidence that future research should examine whether fentanyl predominance in the drug supply may be associated with increases in the optimal buprenorphine dose for treatment of OUD. The currently recommended dose of 16 mg^8,9,10^ was derived from studies conducted prior to the widespread availability of fentanyl,^2,31^ and our finding suggests that a higher dose may now improve retention in treatment. This interpretation is consistent with addiction specialist commentaries that have challenged the value of 16 mg as an optimal buprenorphine dose,^12,13,14^ as well as limited observational analyses suggesting that higher doses may potentially improve buprenorphine treatment effectiveness.^20,21,22,23,24,25,26,27,28^ The findings also support clinician accounts that higher than recommended buprenorphine doses are increasingly needed to suppress withdrawal and cravings.^16^

Preclinical studies suggest that fentanyl downregulates mu opioid receptor expression to a higher degree than morphine and induces greater (or at least more rapid) tolerance to its effects.^18,19^ Assuming that such preclinical data reflect the human condition, it would be predictable that higher doses of a partial agonist like buprenorphine may be needed to substitute for fentanyl than were previously needed to replace opioids like morphine. It may also be worth noting that some emerging benzimidazole opioids (commonly known as nitazenes^32^) are more potent than fentanyl, and are also more efficacious in stimulation of mu opioid receptor signal transduction than fentanyl, suggesting they may also require higher doses of buprenorphine than when morphinans were predominant.^33^

Even at a 24 mg daily dose, 53% of patients in the present study discontinued buprenorphine treatment within 180 days of initiation. This is consistent with previous observational analyses suggesting dose-dependent benefits of daily doses up to 32 mg.^20,21,22,23,24,25,26,27,28^ While some reasons for discontinuation may be unaffected by higher buprenorphine doses,^34^ some patients who use fentanyl require buprenorphine doses greater than the FDA-approved 16 or 24 mg doses to control withdrawal and cravings.^35^ We had hoped to examine doses higher than 24 mg in the current study, but an insufficient number of patients in Rhode Island were prescribed such doses during the study period. This is an important area for future research, as it is uncertain where the ceiling is on the treatment effect in the era of fentanyl.^35^ In a study from before fentanyl’s predominance, retention in treatment increased between 8 and 16 mg doses,^22^ but in our study, retention was similar for doses of 8 and 16 mg. This may suggest a rightward shift in the dose-response curve, which would support reassessment of the treatment effect dose plateau in contemporary cohorts.

### Limitations

This study was observational and, although we attempted to account for measured factors that may be associated with buprenorphine dose and retention in treatment, residual confounding may remain. In particular, clinician prescribing practices may be influenced by unmeasured patient sociodemographic characteristics, social determinants of health (eg, unstable housing^34^), or clinical factors (eg, illicit drug use history, continued use of nonprescribed fentanyl, or comorbid chronic pain). Characteristics of the clinicians’ facilities may also confound the results (eg, addiction clinics with wraparound services vs other settings). These factors could have affected our study, but our findings do support conduct of future randomized clinical trials to examine of the value of higher buprenorphine doses, which may ultimately inform updates to OUD treatment guidelines. Detailed measurement of sociodemographic characteristics, social determinants of health, and clinical factors in such trials would be useful. Furthermore, while this study provides data regarding dose effects on treatment retention, it will also be important to evaluate higher buprenorphine doses in terms of other health outcomes associated with recovery, such as opioid overdose and quality of life. Prior work suggests that improved retention in buprenorphine treatment reduces risk of overdose and death,^3,5,7^ but measurement of these outcomes in a randomized clinical trial would be optimal to evaluate the full risks and benefits of higher buprenorphine doses.

This study also had other important limitations. The Rhode Island PDMP includes data on buprenorphine prescriptions dispensed to Rhode Island residents by out-of-state retail pharmacies but does not capture data on buprenorphine provided in some health care settings (eg, hospital or opioid treatment program) or correctional institutions. In addition, although all patients included in the study had at least 180 days prior to initiation with no buprenorphine dispensed by a retail pharmacy, some may have had buprenorphine treatment prior to April 2016. Classifications of daily dose and treatment discontinuation were based on fill dates, total quantity dispensed, and days’ supply dispensed for each prescription, which may not reflect how the patient took or was instructed to take the medication, especially for the initial prescription. Thus, it was notable that our findings were similar when we considered initial daily dose and that on day 30, when initial titration to a stable dose has typically been completed. We assessed retention in treatment over a 180-day period as to align with the National Quality Forum measure of treatment continuity for OUD.^36^ This measure and our analysis assume all patients benefit from continued buprenorphine treatment for at least 180 days, which may or may not be true for everyone. In addition, we observed improved retention among patients prescribed 24 mg vs 16 mg of buprenorphine, but limitations in the available data prevented assessment of whether 32 mg would be associated with further improvements in retention. Despite these caveats, our longitudinal study design and consideration of a statewide population provide strong support that a rigorous reassessment of optimal buprenorphine dosing is warranted, particularly given fentanyl predominance in the illicit drug supply.

## Conclusions

In this study, during a period of widespread fentanyl availability, a buprenorphine dose of 24 mg was associated with improved retention in treatment compared with the FDA-recommend 16 mg dose. Provided these findings are replicated by future randomized clinical trials, treatment guidelines for OUD may need to be reevaluated to include options for higher buprenorphine doses.

## Notes

Supplement 1. **eTable.** Characteristics of the Study Sample, Among Patients Engaged in Treatment for at Least 30 Days and by Daily Dose on Day 30 (Stability Analysis) **eFigure 1.** Time to Buprenorphine Treatment Discontinuation in the 180 Days After Initiation, Among Patients Engaged in Treatment for at Least 30 Days and by Daily Dose on Day 30 (Stability Analysis) **eFigure 2.** Time to Buprenorphine Treatment Discontinuation in the 180 Days After Initiation, by Daily Dose on Day 0 (Primary Analysis) Including 8 mg

Supplement 2.
Data Sharing Statement

## References

[1] Ahmad FB, Cisewski JA, Rossen LM, Sutton P. Provisional Drug Overdose Death Counts. National Center for Health Statistics. February 15, 2023. Accessed August 18, 2023. https://www.cdc.gov/nchs/nvss/vsrr/drug-overdose-data.htm

[2] Mattick RP, Breen C, Kimber J, Davoli M. Buprenorphine maintenance versus placebo or methadone maintenance for opioid dependence. Cochrane Database Syst Rev. 2014;(2):CD002207. doi:10.1002/14651858.CD002207.pub4 24500948 PMC10617756

[3] Sordo L, Barrio G, Bravo MJ, . Mortality risk during and after opioid substitution treatment: systematic review and meta-analysis of cohort studies. BMJ. 2017;357:j1550. doi:10.1136/bmj.j1550 28446428 PMC5421454

[4] Edelman EJ, Chantarat T, Caffrey S, . The impact of buprenorphine/naloxone treatment on HIV risk behaviors among HIV-infected, opioid-dependent patients. Drug Alcohol Depend. 2014;139:79-85. doi:10.1016/j.drugalcdep.2014.03.006 24726429 PMC4029496

[5] Wakeman SE, Larochelle MR, Ameli O, . Comparative effectiveness of different treatment pathways for opioid use disorder. JAMA Netw Open. 2020;3(2):e1920622. doi:10.1001/jamanetworkopen.2019.20622 32022884 PMC11143463

[6] Weiss L, Botsko M, Egan JE, . Integrating Buprenorphine Treatment Into HIV Clinical Care. Abstract presented at: XVII International AIDS Conference; August 3-8, 2008; Mexico City, Mexico.

[7] Larochelle MR, Bernson D, Land T, . Medication for opioid use disorder after nonfatal opioid overdose and association with mortality: a cohort study. Ann Intern Med. 2018;169(3):137-145. doi:10.7326/M17-3107 29913516 PMC6387681

[8] Sublingual Film SUBOXONE. Prescribing information. Reckitt Benckiser Pharmaceuticals Inc; 2010. Accessed August 18, 2023. https://www.accessdata.fda.gov/drugsatfda_docs/label/2010/022410s000lbl.pdf

[9] Sublingual Tablets SUBOXONE. Prescribing information. Reckitt Benckiser Pharmaceuticals Inc; 2018. Accessed August 18, 2023. https://www.accessdata.fda.gov/drugsatfda_docs/label/2018/020733s022lbl.pdf

[10] Sublingual Tablets SUBUTEX. Prescribing information. Indivior Inc; 2018. Accessed August 18, 2023. https://www.accessdata.fda.gov/drugsatfda_docs/label/2018/020732s018lbl.pdf

[11] Greenwald MK, Herring AA, Perrone J, Nelson LS, Azar P. A neuropharmacological model to explain buprenorphine induction challenges. Ann Emerg Med. 2022;80(6):509-524. doi:10.1016/j.annemergmed.2022.05.032 35940992

[12] Antoine D, Huhn AS, Strain EC, . Method for successfully inducting individuals who use illicit fentanyl onto buprenorphine/naloxone. Am J Addict. 2021;30(1):83-87. doi:10.1111/ajad.13069 32572978 PMC7755703

[13] Bisaga A. What should clinicians do as fentanyl replaces heroin? Addiction. 2019;114(5):782-783. doi:10.1111/add.14522 30661265

[14] Silverstein SM, Daniulaityte R, Martins SS, Miller SC, Carlson RG. “Everything is not right anymore”: Buprenorphine experiences in an era of illicit fentanyl. Int J Drug Policy. 2019;74:76-83. doi:10.1016/j.drugpo.2019.09.003 31563098 PMC6914257

[15] Baca-Atlas MH, Williams JB. Treatment of opioid use disorder attributed to fentanyl with high-dose buprenorphine: a case report. J Clin Psychopharmacol. 2021;41(1):83-85. doi:10.1097/JCP.0000000000001308 33105170

[16] Danilewitz M, McLean M. High-dose buprenorphine for treatment of high potency opioid use disorder. Drug Alcohol Rev. 2020;39(2):135-137. doi:10.1111/dar.13017 31769109

[17] McPherson J, Rivero G, Baptist M, . μ-Opioid receptors: correlation of agonist efficacy for signalling with ability to activate internalization. Mol Pharmacol. 2010;78(4):756-766. doi:10.1124/mol.110.066613 20647394 PMC2981392

[18] Gillis A, Gondin AB, Kliewer A, . Low intrinsic efficacy for G protein activation can explain the improved side effect profiles of new opioid agonists. Sci Signal. 2020;13(625):eaaz3140. doi:10.1126/scisignal.aaz3140 32234959

[19] Kliewer A, Schmiedel F, Sianati S, . Phosphorylation-deficient G-protein-biased μ-opioid receptors improve analgesia and diminish tolerance but worsen opioid side effects. Nat Commun. 2019;10(1):367. doi:10.1038/s41467-018-08162-1 30664663 PMC6341117

[20] Grande LA, Cundiff D, Greenwald MK, Murray M, Wright TE, Martin SA. Evidence on buprenorphine dose limits: a review. J Addict Med. 2023; Epub Ahead of Print. doi:10.1097/ADM.0000000000001189 37788601 PMC10547105

[21] Pizzicato LN, Hom JK, Sun M, Johnson CC, Viner KM. Adherence to buprenorphine: an analysis of prescription drug monitoring program data. Drug Alcohol Depend. 2020;216:108317. doi:10.1016/j.drugalcdep.2020.108317 33035714

[22] Hser YI, Saxon AJ, Huang D, . Treatment retention among patients randomized to buprenorphine/naloxone compared to methadone in a multi-site trial. Addiction. 2014;109(1):79-87. doi:10.1111/add.12333 23961726 PMC3947022

[23] Kakko J, Grönbladh L, Svanborg KD, . A stepped care strategy using buprenorphine and methadone versus conventional methadone maintenance in heroin dependence: a randomized controlled trial. Am J Psychiatry. 2007;164(5):797-803. doi:10.1176/ajp.2007.164.5.797 17475739

[24] Johnson RE, Strain EC, Amass L. Buprenorphine: how to use it right. Drug Alcohol Depend. 2003;70(2)(suppl):S59-S77. doi:10.1016/S0376-8716(03)00060-7 12738351

[25] Leonardi C, Hanna N, Laurenzi P, Fagetti R; I.D.A.C. Group. Multi-centre observational study of buprenorphine use in 32 Italian drug addiction centres. Drug Alcohol Depend. 2008;94(1-3):125-132. doi:10.1016/j.drugalcdep.2007.10.017 18162330

[26] US Food and Drug Administration. NDA 22-410 Suboxone (Buprenorphine/Naloxone) Sublingual Film. 2009. Accessed August 18, 2023. https://www.accessdata.fda.gov/drugsatfda_docs/nda/2010/022410Orig1s000MedR.pdf

[27] Fareed A, Vayalapalli S, Casarella J, Drexler K. Effect of buprenorphine dose on treatment outcome. J Addict Dis. 2012;31(1):8-18. doi:10.1080/10550887.2011.642758 22356665

[28] Daitch D, Daitch J, Novinson D, Frey M, Mitnick C, Pergolizzi J Jr. Conversion from high-dose full-opioid agonists to sublingual buprenorphine reduces pain scores and improves quality of life for chronic pain patients. Pain Med. 2014;15(12):2087-2094. doi:10.1111/pme.12520 25220043

[29] US Drug Enforcement Administration. National Forensic Laboratory Information System - Drug 2021 Annual Report. 2022. Accessed August 18, 2023. https://www.nflis.deadiversion.usdoj.gov/nflisdata/docs/NFLISDrug_2021AnnualReport.pdf.

[30] Hallowell BD, Weidele HR, Scagos RP. Accidental drug overdose deaths in Rhode Island: January 1, 2016-July 31, 2020. R I Med J (2013). 2020;103(10):62-65. 33261239

[31] Jacobs P, Ang A, Hillhouse MP, . Treatment outcomes in opioid dependent patients with different buprenorphine/naloxone induction dosing patterns and trajectories. Am J Addict. 2015;24(7):667-675. doi:10.1111/ajad.12288 26400835 PMC5322942

[32] Hasegawa K, Minakata K, Suzuki M, Suzuki O. Non-fentanyl-derived synthetic opioids emerging during recent years. Forensic Toxicol. 2022;40(2):234-243. doi:10.1007/s11419-022-00624-y PMC9052731 35528111

[33] Vandeputte MM, Van Uytfanghe K, Layle NK, St Germaine DM, Iula DM, Stove CP. Synthesis, chemical characterization, and μ-opioid receptor activity assessment of the emerging group of “nitazene” 2-benzylbenzimidazole synthetic opioids. ACS Chem Neurosci. 2021;12(7):1241-1251. doi:10.1021/acschemneuro.1c00064 33759494

[34] Godersky ME, Saxon AJ, Merrill JO, Samet JH, Simoni JM, Tsui JI. Provider and patient perspectives on barriers to buprenorphine adherence and the acceptability of video directly observed therapy to enhance adherence. Addict Sci Clin Pract. 2019;14(1):11. doi:10.1186/s13722-019-0139-3 30867068 PMC6417248

[35] Herring AA, Vosooghi AA, Luftig J, . High-dose buprenorphine induction in the emergency department for treatment of opioid use disorder. JAMA Netw Open. 2021;4(7):e2117128. doi:10.1001/jamanetworkopen.2021.17128 34264326 PMC8283555

[36] US Centers for Medicare & Medicaid Services. Quality ID #468 (NQF 3175): Continuity of Pharmacotherapy for Opioid Use Disorder (OUD). 2019. Accessed August 18, 2023. https://qpp.cms.gov/docs/QPP_quality_measure_specifications/CQM-Measures/2019_Measure_468_MIPSCQM.pdf

