# Supplementary material for: Buprenorphine Dose and Time to Discontinuation Among Patients With Opioid Use Disorder in the Era of Fentanyl
Source: JAMA Netw Open. 2023 Sep 18;6(9):e2334540. doi: 10.1001/jamanetworkopen.2023.34540 (PMC10507490; doi:10.1001/jamanetworkopen.2023.34540)
Supplement: Supplement 1. — eTable. Characteristics of the Study Sample, Among Patients Engaged in Treatment for at Least 30 Days and by Daily Dose on Day 30 (Stability Analysis) eFigure 1. Time to Buprenorphine Treatment Discontinuation in the 180 Days After Initiation, Among Patients Engaged in Treatment for at Least 30 Days and by Daily Dose on Day 30 (Stability Analysis) eFigure 2. Time to Buprenorphine Treatment Discontinuation in the 180 Days After Initiation, by Daily Dose on Day 0 (Primary Analysis) Including 8 mg [file jamanetwopen-e2334540-s001.pdf]

Supplemental Online Content

Chambers LC, Hallowell BD, Zullo AR, et al. Buprenorphine dose and time to discontinuation among patients with opioid use disorder in the era of fentanyl. *JAMA Netw Open.* 2023;6(9):e2334540. doi:10.1001/jamanetworkopen.2023.34540

eTable. Characteristics of the Study Sample, Among Patients Engaged in Treatment for At Least 30 Days and By Daily Dose on Day 30 (Stability Analysis)

eFigure 1. Time to Buprenorphine Treatment Discontinuation in the 180 Days After Initiation, Among Patients Engaged in Treatment for At Least 30 Days and By Daily Dose on Day 30 (Stability Analysis)

eFigure 2. Time to Buprenorphine Treatment Discontinuation in the 180 Days After Initiation, By Daily Dose on Day 0 (Primary Analysis) Including 8 mg

This supplemental material has been provided by the authors to give readers additional information about their work.

© 2023 Chambers LC et al. *JAMA Network Open*.

eTable. Sociodemographic characteristics, prescription history in prior 30 days, and buprenorphine prescription characteristics on day 30 of Rhode Island residents initiating buprenorphine treatment for OUD between October 1, 2016, and September 30, 2020, and engaging in treatment for at least 30 days, overall and by daily dose prescribed on day 30 (16 mg versus 24 mg, stability analysis).

	**Overall** N=4,318 n (%)	**Daily dose on day 30*** **16 mg** N=1,916 n (%)	**24 mg** N=817 n (%)	**P-value†**
**Sociodemographic characteristics**				
Age group (years)				
18-24	247 (6)	122 (6)	23 (3)	**<0.001**
25-34	1,269 (29)	588 (31)	227 (28)	
35-44	1,210 (28)	540 (28)	243 (30)	
45-54	847 (20)	372 (19)	176 (22)	
≥55	745 (17)	294 (15)	148 (18)	
Sex assigned at birth				
Female	1,645 (38)	679 (35)	291 (36)	0.07
Male	2,607 (60)	1,193 (62)	518 (63)	
Unknown	66 (2)	44 (2)	8 (1)	
Health insurance type				
Medicaid	1,498 (35)	661 (35)	305 (37)	**0.04**
Medicare	429 (10)	179 (9)	96 (12)	
Private	1,941 (45)	893 (47)	354 (43)	
Other or none	450 (10)	183 (10)	62 (8)	
Year of treatment initiation				
2016	371 (9)	194 (10)	54 (7)	**0.001**
2017	1,274 (30)	609 (32)	227 (28)	
2018	1,235 (29)	544 (28)	226 (28)	
2019	864 (20)	351 (18)	198 (24)	
2020	574 (13)	218 (11)	112 (14)	
**Prescription history in prior 30 days**				
Available days' supply of a benzodiazepine				
Yes	687 (16)	271 (14)	153 (19)	**0.002**
No	3,631 (84)	1,645 (86)	664 (81)	
Available days' supply of an opioid other than buprenorphine				
Yes	730 (17)	303 (16)	164 (20)	**0.007**
No	3,588 (83)	1,613 (84)	653 (80)	
**Buprenorphine prescription characteristics on day 30**				
Product type				
Buprenorphine monoproduct	255 (6)	82 (4)	43 (5)	0.26
Buprenorphine/naloxone	4,063 (94)	1,834 (96)	774 (95)	
Product formulation				
Film	2,685 (62)	1,189 (62)	508 (62)	0.95
Tablet	1,633 (37)	727 (38)	309 (38)	
Days' supply (days)				

© 2023 Chambers LC et al. *JAMA Network Open*.

<8	1,329 (31)	662 (35)	247 (30)	**0.03**
≥8	2,989 (69)	1,254 (65)	570 (70)	
Distance from home to pharmacy (miles)‡				
<5	3,185 (74)	1,392 (73)	620 (76)	0.18
≥5	1,122 (26)	519 (27)	194 (24)	
Unknown	11 (<1)	5 (<1)	<5§	

Abbreviation: OUD, opioid use disorder.
* Defined as the daily dose prescribed on day 30, based on the fill dates and total quantity and days' supply dispensed; if no prescription covered day 30, then the most recent prior daily dose was used.
† Bold indicates a p-value of <0.05.
‡ Based on ZIP code centroids.
§ Counts of 1-4 and associated percentages are suppressed, in accordance with the Small Numbers Policy of the Rhode Island Department of Health.

© 2023 Chambers LC et al. *JAMA Network Open*.

eFigure 1. Inverse probability weighted time to buprenorphine treatment discontinuation* in the 180 days after initiation, among patients who engaged in treatment for at least 30 days, by daily

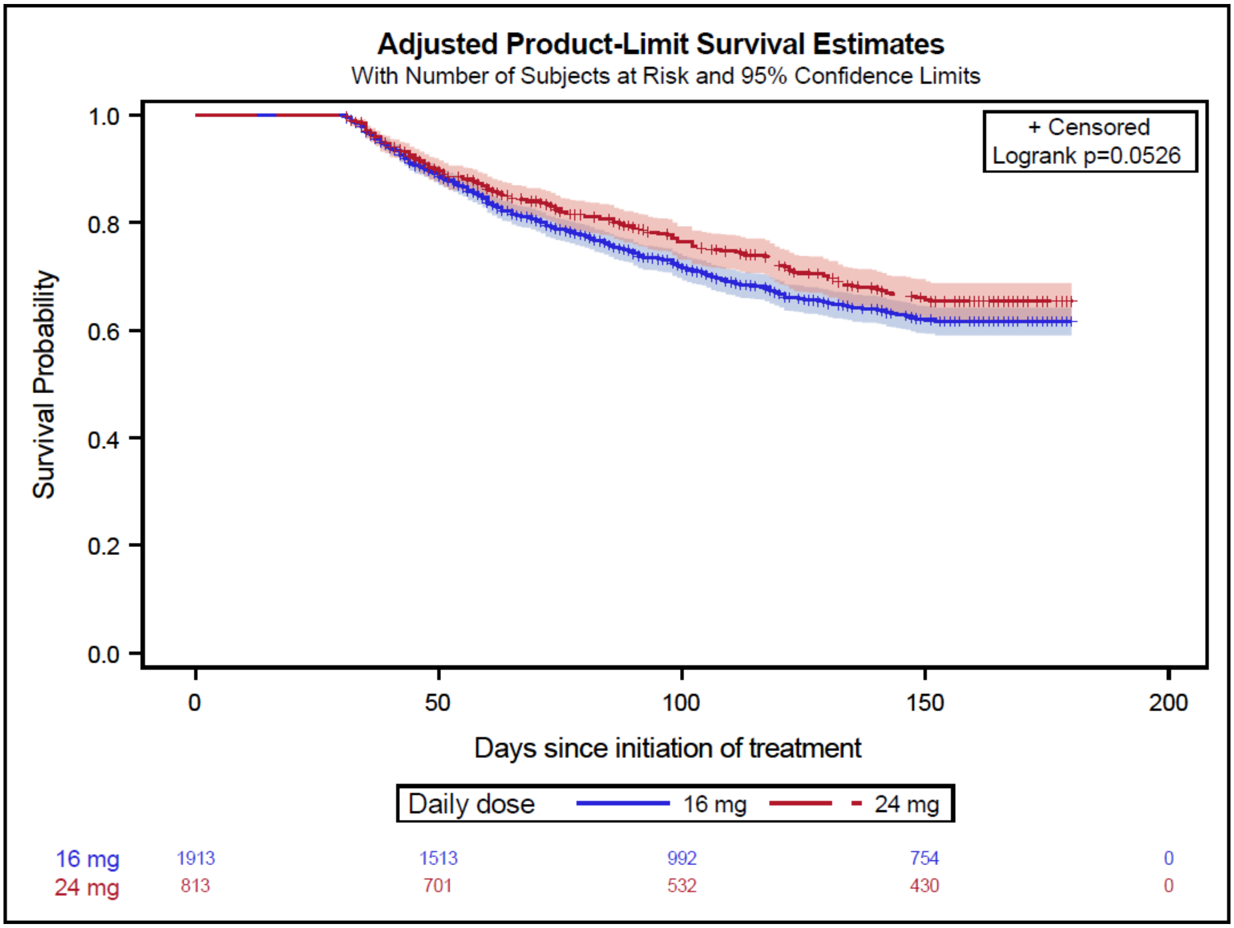

dose prescribed on day 30 (stability analysis).

Notes: Survival probability indicates the probability patients are retained in buprenorphine treatment, with the shaded area representing the 95% confidence interval. The number of patients at risk over time has been re-weighted and, thus, is not expected to align with the overall study sample size.
* Defined as a gap in treatment of more than 27 days, based on prescription fill dates and days' supply. Controls for potential informative censoring using stabilized inverse probability of censoring weights and for potential confounding using stabilized inverse probability of treatment weights.

© 2023 Chambers LC et al. *JAMA Network Open*.

eFigure 2. Inverse probability weighted time to buprenorphine treatment discontinuation* in the

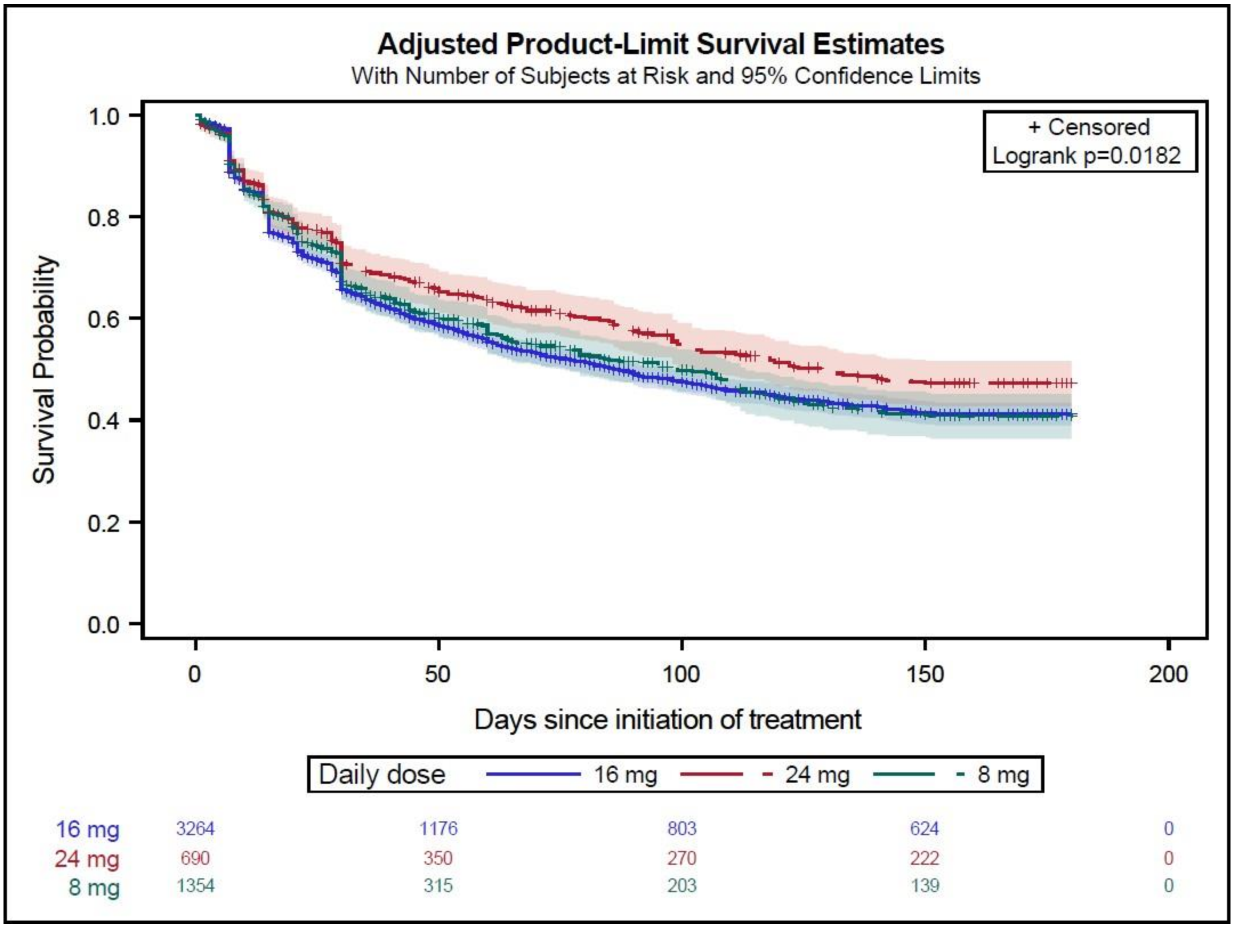

180 days after initiation, by daily dose starting on day 0 (primary analysis) and including 8 mg.

Notes: Survival probability indicates the probability patients are retained in buprenorphine treatment, with the shaded area representing the 95% confidence interval. The number of patients at risk over time has been re-weighted and, thus, is not expected to align with the overall study sample size.

* Defined as a gap in treatment of more than 27 days, based on prescription fill dates and days' supply. Controls for potential informative censoring using stabilized inverse probability of censoring weights and for potential confounding using stabilized inverse probability of treatment weights.

© 2023 Chambers LC et al. *JAMA Network Open*.
