# Supplementary material for: Buprenorphine Dose and Time to Discontinuation Among Patients With Opioid Use Disorder in the Era of Fentanyl
Source: JAMA Netw Open. 2023 Sep 18;6(9):e2334540. doi: 10.1001/jamanetworkopen.2023.34540 (PMC10507490; doi:10.1001/jamanetworkopen.2023.34540)
Supplement: Supplement 2. — Data Sharing Statement [file jamanetwopen-e2334540-s002.pdf]

Data Sharing Statement

Chambers. Buprenorphine Dose and Time to Discontinuation Among Patients With Opioid Use Disorder in the Era of Fentanyl. *JAMA Netw Open*. Published September 18, 2023. doi:10.1001/jamanetworkopen.2023.34540

Data
Data available: No

Additional Information
Explanation for why data not available: We used data from the Rhode Island Department of Health, which are subject to the data sharing policies and procedures of the agency.
